# L2 β-lactamase contributes to ceftolozane-tazobactam resistance in *Pseudomonas aeruginosa*

**DOI:** 10.1128/aac.00356-26

**Published:** 2026-09-04

**Authors:** Preeti Garai, Sophia H. Nozick, Caroline C. Jozefczyk, Hannah Nam, J. Nicholas O’Donnell, Egon A. Ozer, Alan R. Hauser, Nathaniel J. Rhodes

**Affiliations:** 1 Department of Microbiology-Immunology, Northwestern University, Feinberg School of Medicine 547641, Chicago, Illinois, USA; 2 Midwestern University College of Pharmacy, Downers Grove Campus 15475, Downers Grove, Illinois, USA; 3 Department of Pharmacy, Northwestern Medicine 370076, Chicago, Illinois, USA; 4 Stanford University School of Medicine 10624, Stanford, California, USA; 5 Infectious Diseases, University of California Irvine 12219, Orange, California, USA; 6 Department of Pharmacy Practice, Albany College of Pharmacy and Health Sciences 1091, Albany, New York, USA; 7 Division of Infectious Diseases, Department of Medicine, Northwestern University, Feinberg School of Medicine 12244, Chicago, Illinois, USA; 8 Department of Pharmacy Practice, Midwestern University College of Pharmacy Downers Grove Campus 15475, Downers Grove, Illinois, USA; 9 Pharmacometrics Center of Excellence, Midwestern University College of Pharmacy, Downers Grove Campus 15475, Downers Grove, Illinois, USA; Entasis, Big Bay, Michigan, USA

**Keywords:** ceftolozane-tazobactam, resistance, whole-genome sequencing, bioinformatics, β-lactamases

## Abstract

The prevalence of non-susceptibility to ceftolozane-tazobactam (C/T) among *Pseudomonas aeruginosa* remains low, but novel mechanisms of C/T resistance are of concern. Herein, we describe a novel *P. aeruginosa* genotype associated with high-level C/T resistance (>256/4 µg/mL) in a single patient. Whole-genome sequencing of the isolate was compared to that of a susceptible isolate cultured from the same patient 2 months earlier. Analysis of the sequences revealed two different *P. aeruginosa* high-risk clones: sequence type (ST)111 followed by ST235. The C/T-resistant ST235 isolate contained five copies of a genetic element composed of an L2 β-lactamase gene (*bla*_L2_) and a truncated *ampR*_L2_ transcriptional regulator gene, which are commonly found together in *Stenotrophomonas maltophilia* strains and have not been reported to mediate resistance to C/T. Comparative genomic analysis with other *P. aeruginosa* isolates failed to identify alternative explanations for the observed C/T resistance. We found that exogenous expression of *bla*_L2_ increased C/T minimum inhibitory concentrations (MICs) in genetically distinct *P. aeruginosa* strains. A screen of our archived isolates identified two *P. aeruginosa* clinical isolates, PS2045 and PS2046, with one and two copies, respectively, of the genetic element containing *bla*_L2_ and truncated *ampR*_L2_. Interestingly, disruption of the gene *bla*_L2_ but not the truncated *ampR*_L2_ in PS2045 led to a decrease in C/T MIC. Thus, we report a novel mechanism of C/T resistance in *P. aeruginosa* partially mediated by an L2 β-lactamase independently of its canonical regulator, AmpR_L2_.

## INTRODUCTION

Rising rates of antibiotic resistance in the bacterial pathogen *Pseudomonas aeruginosa* pose a serious threat to public health. Infections caused by antibiotic-resistant strains of *P. aeruginosa* are extremely difficult to treat and are associated with severe morbidity and high mortality rates. Ceftolozane-tazobactam (C/T), a combination of an antipseudomonal cephalosporin, ceftolozane, and a β-lactamase inhibitor, tazobactam (1, 2), has been efficacious in treating *P. aeruginosa* infections (2–5). The high activity of C/T against multidrug resistance, extensive drug resistance, and difficult-to-treat resistance among strains of *P. aeruginosa* (2, 6–10) is attributable to the ability of C/T to evade several antibiotic resistance mechanisms employed by *P. aeruginosa* that inactivate first-line antipseudomonal antibiotics (11, 12).

The prevalence of *P. aeruginosa* non-susceptibility to C/T remains low among clinical isolates from critically ill patients, ranging from 3% to 18% (4, 6, 8, 13), but the evolution of C/T resistance during therapy is concerning. While the most common mechanism of C/T resistance in *P. aeruginosa* involves mutations leading to overexpression or increased hydrolytic activity of the class C β-lactamase AmpC (14–16), other mechanisms such as the acquisition of extended-spectrum β-lactamase (ESBL) genes and overexpression of efflux pumps have been occasionally reported (15, 17–22). Given the results of the recent multicenter CACTUS study of hospitalized patients in the USA, which found greater clinical efficacy (OR = 2.07, 95% CI 1.16–3.70) with C/T vs ceftazidime-avibactam (CZA) across a variety of *P. aeruginosa* infections (23), it is expected that C/T use and resistance will increase in the near term. Thus, it is crucial to understand emerging C/T resistance mechanisms in *P. aeruginosa*. Herein, we describe a novel mechanism of C/T resistance partially mediated by acquisition of the L2 β-lactamase gene (*bla*_L2_) by *P. aeruginosa*.

## MATERIALS AND METHODS

### Patient data collection

All clinical data were obtained from the Northwestern Memorial Hospital (NMH), Chicago, electronic medical records.

### Microbiology and antibiotic susceptibility testing

All strains used in this study are described in Table 1. Antibiotic susceptibility testing was performed by microbroth dilution (MBD) using a protocol (24) adapted from Clinical and Laboratory Standards Institute guidance or commercially available Sensititre plates (Thermo Fisher Scientific). C/T minimum inhibitory concentrations (MICs) were also determined using commercially available MIC test strip (MTS) assays (Liofilchem). Details of methods used are included in Supplemental Methods.

**TABLE 1:** Bacterial strains used in this study^*a*^

Species	Strain	Characteristics	Source (reference)	GenBank accession number
*P. aeruginosa*	PA-NM-055	Collected from endotracheal tube aspirate	This study, NMH	JBUYFB000000000.1
*P. aeruginosa*	PA-NM-086	Collected from bone biopsy	This study, NMH	JBUYFC000000000.1
*P. aeruginosa*	PS2045	Collected from blood	This study, NMH	JAHTGK000000000.2
*P. aeruginosa*	PS2046	Collected from blood	This study, NMH	JAHTGL000000000.2
*P. aeruginosa*	PA14	Reference strain		GCA_000014625.1
*P. aeruginosa*	PA14-pPSV37 (empty vector)	Lab construct, PA14 containing pPSV37 plasmid	This study	NA
*P. aeruginosa*	PA14-p*bla*_L2_	Lab construct, PA14 containing p*bla*_L2_ plasmid	This study	NA
*P. aeruginosa*	PAO1	Reference strain		GCA_000006765.1
*P. aeruginosa*	PAO1-pPSV37 (empty vector)	Lab construct, PAO1 containing pPSV37 plasmid	This study	NA
*P. aeruginosa*	PAO1-p*bla*_L2_	Lab construct, PAO1 containing p*bla*_L2_ plasmid	This study	NA
*P. aeruginosa*	PS2045 ∆*bla*_L2_^act^	Lab construct made using pex18-Hyg^R^	This study	SRR37233608
*P. aeruginosa*	PS2045 ∆*ampR*_L2_^tr^	Lab construct made using pex18-Hyg^R^	This study	SRR37233606
*E. coli*	Top10	F- *mcrA* Δ(*mrr-hsdRMS-mcrBC*) φ80*lacZ*ΔM15 Δ*lacX74 nupG recA1* *araD139* Δ(*ara-leu*)7697 *galE15 galK16 rpsL*(StrR) *endA1* λ-	Invitrogen	NA
*E. coli*	SM10 λpir	TpR SmR *recA*, *thi*, *pro*, *hsdR*-M + RP4: 2-Tc:Mu: Km Tn7 λpir	(25)	NA

^*a*^ Bacterial strains used in the study are listed along with their characteristics and sources. GenBank accession numbers for the strains collected in this study and reference strains are provided. ‘NA’ indicates sequences not available. References are provided in parentheses. NMH, Northwestern Memorial Hospital.

### Whole-genome sequencing and bioinformatics

Overnight cultures in LB broth grown from single colonies of isolates streaked onto LB agar were used for genomic DNA isolation. Libraries were prepared from genomic DNA using Nextera XT DNA Library Preparation Kit (Illumina) and the Oxford Nanopore Ligation Sequencing Kit (Nanopore) for whole-genome sequencing (WGS) by short-read (Illumina) and long-read sequencing (MinION), respectively. Additional details on WGS and bioinformatics are contained in Supplemental Methods.

### Phylogenetic analysis

All publicly available *P. aeruginosa* genome assemblies in the NCBI assembly database were used to characterize the prevalence of *bla*_L2_ among *P. aeruginosa* genomes using nucleotide BLAST, sequence alignment, phylogenetic analysis, and tree visualization (26). Whole-genome phylogenetic relationships were estimated for all sequence type (ST)235 *P. aeruginosa* genomes using Mashtree v1.2.0 (27). Details of the phylogenetic analysis are provided in Supplemental Methods.

### Construction of strains expressing *bla*_L2_

The strains PA14 and PAO1 were genetically modified to exogenously express *bla*_L2_ (Table 1). Details of strain construction are included in Supplemental Methods. Briefly, *bla*_L2_ was amplified from the genomic DNA of PA-NM-086 and integrated downstream to an isopropyl-β-D-thiogalactopyranoside (IPTG)-inducible *lac*UV5 promoter in the plasmid pPSV37-Gen^R^ (28) (Table S1). The resulting recombinant plasmid (“p*bla*_L2_”) was introduced into the *P. aeruginosa* strains via conjugation with *Escherichia coli* strain SM10 (25) and verified by Sanger sequencing. Vector control strains containing the empty vector (EV) pPSV37 were generated using the method described above.

### RNA extraction and reverse transcriptase-quantitative PCR

Bacterial cultures grown until late-log phase in LB broth were treated with IPTG (1 mM) or imipenem (1 µg/mL) for 2 hours and processed for RNA extraction (Qiagen) and cDNA synthesis (Invitrogen). Negative control cultures were left untreated. Reverse transcriptase-quantitative PCR (RT-qPCR) was performed using qPCR probe assays (Integrated DNA Technologies) on a CFX-96 Real-Time Thermocycler (Bio-Rad). The expression level of target genes, after normalizing to that of the housekeeping gene *rpoD*, was plotted as abundance in RNA level relative to the control strain, PA14. Additional details of sample preparation, processing, and analysis are described in Supplemental Methods.

### Generation of deletion mutants

A suicide vector-mediated allelic replacement method (29) was used for the targeted deletion of a 477 bp internal segment of *bla*_L2_, which encodes the S-X-X-K motif and SDN loop of the L2 β-lactamase (30), and full length of *ampR*_L2_^tr^ in PS2045. Briefly, ~500 bp fragments homologous to regions flanking the target genes were cloned in the plasmid pEX18-Hyg^R^ (Table S1). PS2045 was transformed with the resultant plasmid using the method described above (25) to allow the deletion of the target gene by homologous recombination between the plasmid and the bacterial genome. Each mutant was confirmed by whole-genome sequencing. Details of the method are provided in Supplemental Methods.

### Statistical analysis

Experiments were performed with at least two biological replicates, and results were plotted using GraphPad Prism (v10). Differences in mean RNA abundance level between isolates were determined using one-way ANOVA with Tukey’s post hoc correction. Student’s *t*-test was used for comparing two samples. For all analyses, *P* < 0.05 was considered statistically significant.

## RESULTS

### Patient case

**Fig 1:**
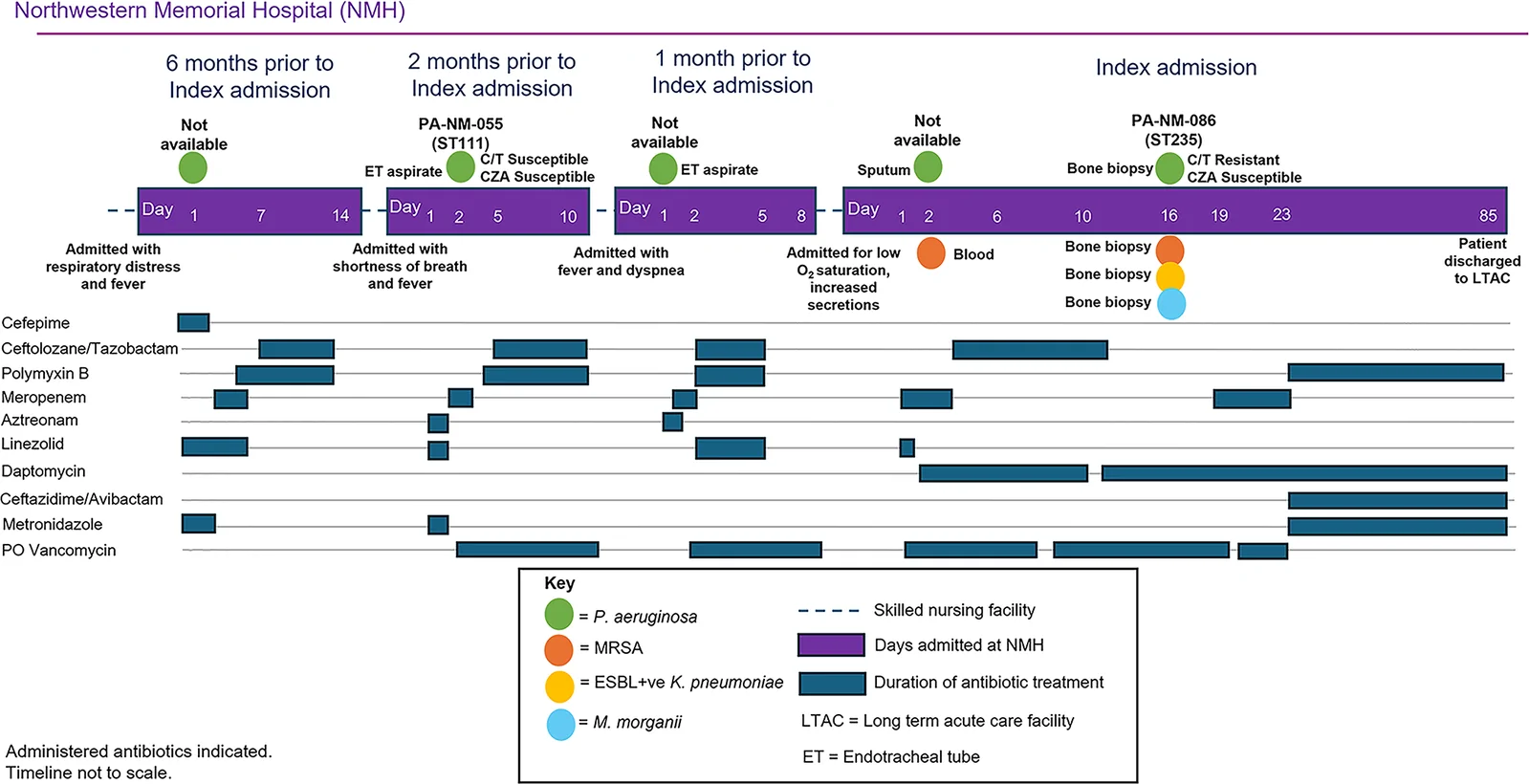
Patient history of infection and antibiotic therapy. The history of the patient’s clinical course at NMH, including admissions, isolates collected, sample sources, and antibiotic therapy, is depicted. *P. aeruginosa* isolates collected during multiple admissions are shown with C/T and CZA susceptibilities for the available isolates above the horizontal bar indicating days of admission.

A 51-year-old male with a complex medical history, including decubitus ulcers complicated by chronic osteomyelitis and chronic respiratory failure with recurrent *P. aeruginosa* pneumonia, was admitted to NMH with reports of low oxygen saturations, increased respiratory secretions, and hyponatremia. Two months prior, he presented with pneumonia caused by *P. aeruginosa* (saved as PA-NM-055) and was treated with C/T plus inhaled and intravenous (IV) polymyxin B (Fig. 1). During the index admission, he was found to have methicillin-resistant *Staphylococcus aureus* (MRSA) in blood cultures and *P. aeruginosa* growing in sputum. Empiric antibiotics were changed to daptomycin plus C/T. His pulmonary symptoms improved, and his subsequent blood cultures were negative; however, he remained intermittently febrile. A sacral bone biopsy grew ESBL-positive *Klebsiella pneumoniae*, *Morganella morganii*, MRSA, and *P. aeruginosa* (saved as PA-NM-086). Daptomycin was continued, and meropenem was added for ESBL coverage of osteomyelitis. Additional susceptibility testing by the clinical microbiology laboratory showed that PA-NM-086 exhibited high-level C/T resistance but susceptibility to CZA (Fig. 1; Table 2). Meropenem was changed to CZA plus IV polymyxin B and metronidazole, with a planned treatment duration of 6 weeks post-biopsy. On hospital day 85, the patient was discharged to an acute inpatient rehabilitation center.

**TABLE 2:** Susceptibility profiles of *P. aeruginosa* isolates used in the study^*a*^

Strain	PA-NM-055	PA-NM-086	PS2046	PS2045	PS2045 ∆*bla*_L2_^act^	PS2045 ∆*ampR*_L2_^tr^	PA14-EV + IPTG	PA14-p*bla*_L2_ + IPTG	PAO1-EV + IPTG	PAO1-p*bla*_L2_ + IPTG
MLST	ST111	ST235	ST235	ST235	ST235	ST235	ST253	ST253	ST549	ST549
PDC (*bla*_AmpC_) variant	PDC3	PDC35	PDC35	PDC35	PDC35	PDC35	PDC34	PDC34	PDC1	PDC1
Number of *bla*_L2_	0	5	2	1	0	1	0	1	0	1
MICs (µg/mL)										
Ceftolozane-tazobactam (C/T)^b^	1/4	256/4	256/4	32/4	**0.5/4**	32/4	0.25/4	**16/4**	0.5/4	**32/4**
Ceftazidime-avibactam (CZA)^c^	≤2/4	≤2/4	≤2/4	≤2/4	≤2/4	≤2/4	≤2/4	≤2/4	≤2/4	≤2/4
Ceftazidime^c^	16	≥32	≥32	16	**≤1**	8	≤1	**8**	≤1	**8**
Cefepime^c^	≥32	≥32	≥32	16	**4**	16	≤2	**8**	≤2	**16**
Aztreonam^c^	8	≥32	≥32	≥32	**4**	≥32	2	**≥32**	4	**≥32**
Imipenem^c^	≥16	≥16	≤1	≤1	≤1	≤1	≤1	≤1	≤1	≤1
Meropenem^c^	8	4	≤0.5	≤0.5	≤0.5	≤0.5	≤0.5	≤0.5	≤0.5	≤0.5
Doripenem^c^	≥8	4	≤0.5	≤0.5	≤0.5	≤0.5	≤0.5	≤0.5	≤0.5	≤0.5
Piperacillin-tazobactam^c^	≥128/4	≥128/4	≥128/4	16/4	≤8/4	16/4	≤8/4	16/4	≤8/4	16/4
Ticarcillin-clavulanic acid^c^	128/2	≥256/2	≥256/2	32/2	32/2	NA	NA	NA	NA	NA

^*a*^ Isolates used in the study are listed with their MLST, PDC (*bla*_AmpC_) variant, copy number of *bla*_L2_ gene, and MICs (µg/mL). MICs for ^b^C/T by microbroth dilution and for ^c^other antibiotics by Sensititre are indicated for each strain. The median or higher of two medians of MIC values from two or more experiments was chosen as the final MIC. The MICs altered by at least two doubling dilutions due to deletion or overexpression of *bla*_L2_ are highlighted in bold. NA indicates results are not available.

### Antibiotic resistance phenotypes of *P. aeruginosa* isolates collected from the patient

The respiratory isolate PA-NM-055 was found to be susceptible to C/T but the bone biopsy isolate PA-NM-086 was resistant (Table 2; Table S3). These observations gave the clinical impression that C/T resistance emerged after repeated courses of this antibiotic. However, genomic analysis revealed that PA-NM-055 was ST111, whereas PA-NM-086 was ST235 (Table 2), indicating infections with two distinct high-risk clones of *P. aeruginosa*.

### Antimicrobial resistance genes in the C/T-resistant strain PA-NM-086

Long-read *de novo* assembly with short-read correction yielded a single circular 6.92 Mb chromosome for PA-NM-086. Examination of the *Pseudomonas*-derived cephalosporinase (PDC) gene sequence, *bla*_AmpC_, a member of the AmpC family, revealed that PA-NM-086 contained the PDC-35 variant, which does not confer C/T resistance (15, 17). Similarly, C/T resistance-associated mutations in the PDC regulator gene (AmpR D135N/G) (15) or efflux pump genes (MexD Q178R, MexD S133G, and MexZ L172P) (21, 31) and ESBLs (OXA-539, GES-6, SHV-2a, and VIM-2) (20, 32) were absent in PA-NM-086. However, PA-NM-086 contained the Ambler class A β-lactamase gene *bla*_L2_, which was not present in the patient’s banked respiratory isolate PA-NM-055.

### Phylogenetic analysis of *bla*_L2_ in *P. aeruginosa*

**Fig 2:**
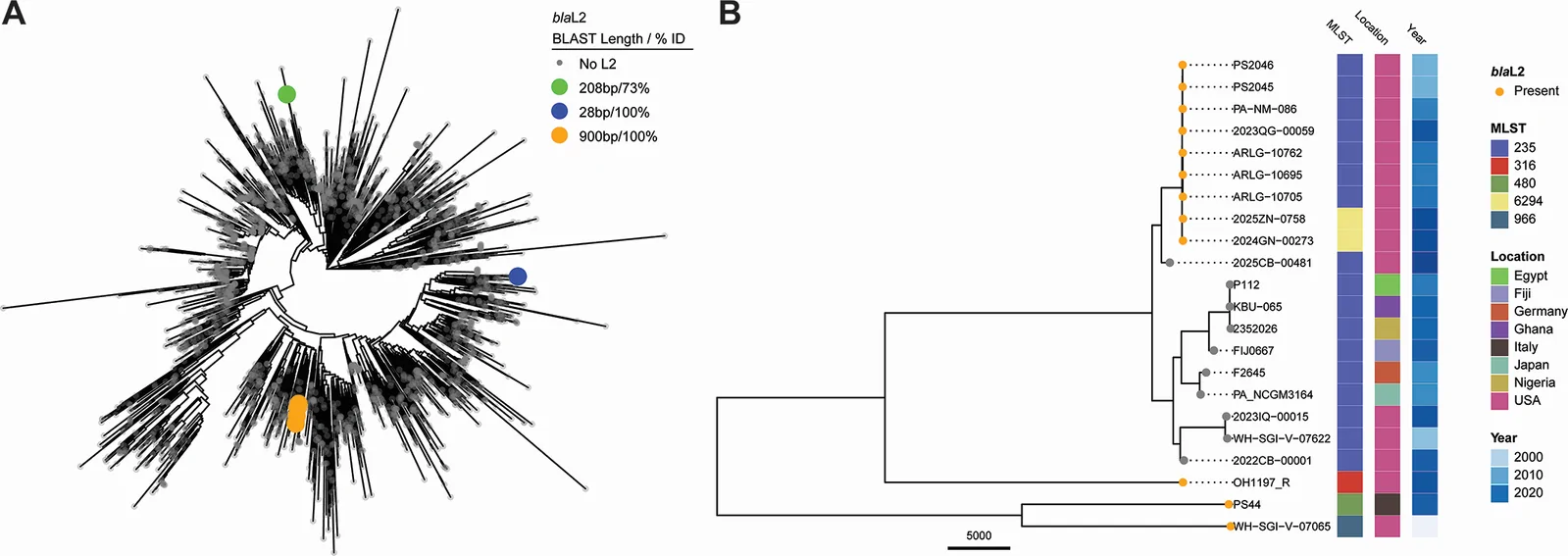
The presence of the *bla*_L2_ gene among publicly available *P. aeruginosa* genomes. (**A**) Whole-genome phylogenetic analysis of ST235 genome assemblies in NCBI (*n* = 2,995, one outlier removed) using Mashtree. Isolates with blastn hits for the *bla*_L2_ gene are highlighted. Taxa are colored by the alignment length and percent identity of the BLAST results. Six genomes had identical genes (orange), and two genomes contained portions of the *bla*_L2_ gene (blue and green). (**B**) Phylogenetic relationships of all *bla*_L2_-positive genome sequences in NCBI by Mashtree analysis. Isolates carrying the *bla*_L2_ gene (orange) with >88% blastn sequence identity across >95% length of the *bla*_L2_ gene and relative to the 10 randomly selected non-*bla*_L2_ ST235 isolates from NCBI (gray) are shown. Bars show the multilocus sequence type (MLST), geographic location, and decade of collection of each isolate.

The *bla*_L2_ gene from PA-NM-086 shared 100% identity with the L2 β-lactamase gene found in many *Stenotrophomonas maltophilia* strains (30). Analysis of *P. aeruginosa* ST235 genomes available in NCBI identified six genomes (0.2% of ST235) containing the *bla*_L2_ gene (Fig. 2A). Across all the *P. aeruginosa* genomes in NCBI, *bla*_L2_ was identified in 12 isolates (0.02% of all *P. aeruginosa*), 11 of which were collected in the USA (Fig. 2B). Furthermore, this gene was predominantly confined to five lineages (Fig. 2B), suggesting that the transfer of *bla*_L2_ from *S. maltophilia* to *P. aeruginosa* was rare.

### Genetic context of *bla*_L2_

**Fig 3:**
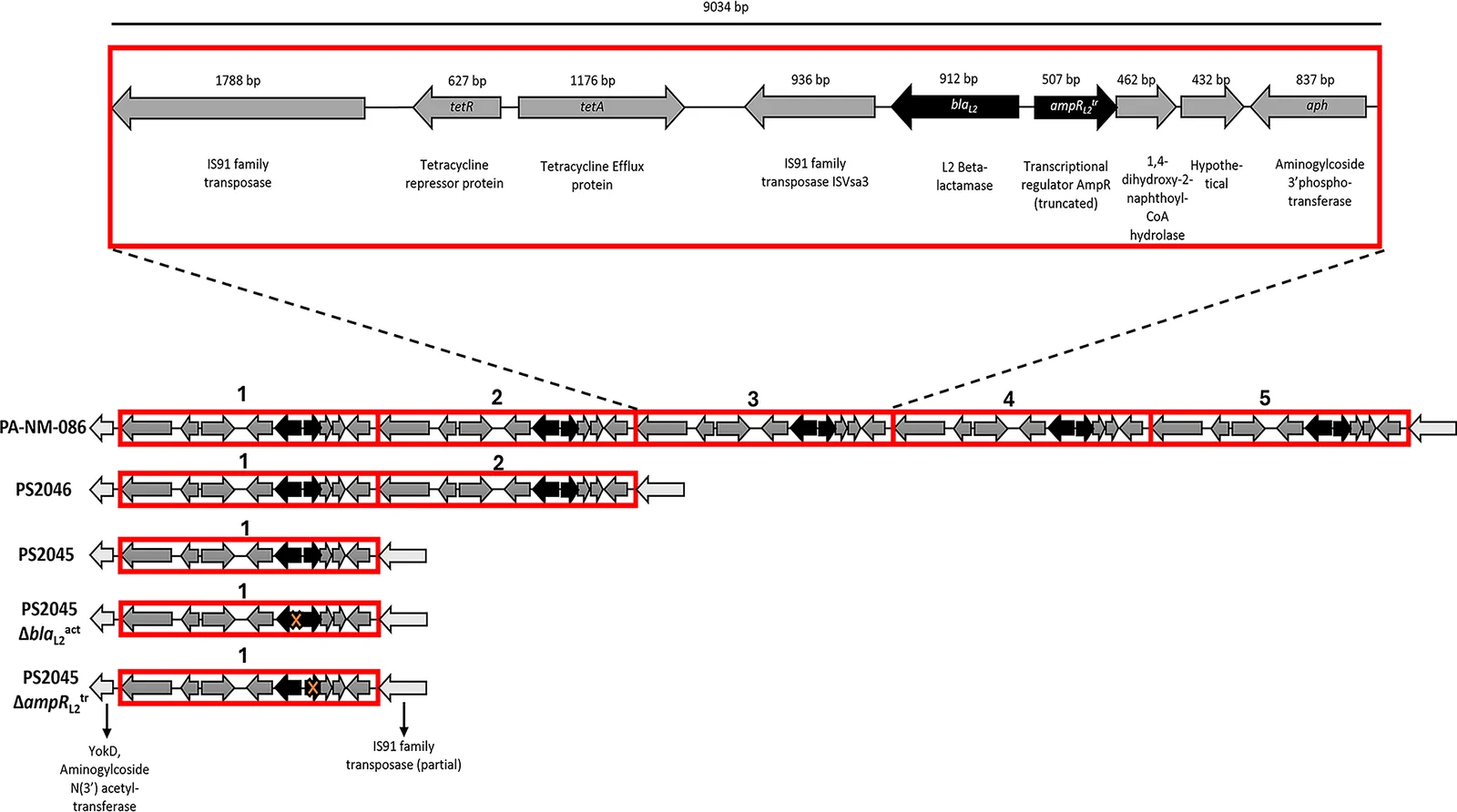
Genomic element containing *bla*_L2_. The genomic element (9034 bp) containing *bla*_L2_ is depicted in the red box at the top, indicating the length and annotation of each open reading frame. The number of copies of this region in the strains PA-NM-086 (five tandem copies), PS2046 (two tandem copies), and PS2045 (one copy), which are always flanked by the same genes, is indicated. The deleted segments in this region in the deletion mutants PS2045 ∆*bla*_L2_^act^ and PS2045 ∆*ampR*_L2_^tr^ are shown as cross marks.

In PA-NM-086, the 912 bp *bla*_L2_ gene was adjacent to an *ampR*_L2_ gene that had a reduced length of 507 bp compared to 867 bp for the *ampR*_L2_ gene of *S. maltophilia*. We designated this truncated *ampR*_L2_ allele “*ampR*_L2_^tr^.” It encodes a predicted protein of 168 amino acids, of which the first 129 amino acids are identical to that of AmpR _L2_ of *S. maltophilia*. Of note, the *ampR* gene directly adjacent to *bla*_AmpC_ (33) (*ampR*_AmpC_) is distinct from *ampR*_L2_ in *S. maltophilia* (71.6% identity over 67% of the *ampR*_AmpC_ gene). The truncated *ampR*_L2_^tr^ gene in PA-NM-086 has only 72% identity over 41% of the *ampR*_AmpC_ gene. In the genome of PA-NM-086, *bla*_L2_ and *ampR*_L2_^tr^ were part of a larger 9,034 bp element that was repeated in tandem five times (Fig. 3). This region also contained two tetracycline resistance genes (*tetR* and *tetA* [34, 35]), an aminoglycoside resistance gene (*aph* [36]), and IS91 family transposases.

### Relationship of *bla*_L2_ copy number and C/T resistance

A search of *P. aeruginosa* WGSs in our collections identified two clinical isolates, PS2045 and PS2046 (Table 1; Fig. 2B), containing one and two identical copies, respectively, of the genetic element containing *bla*_L2_ (Fig. 3). Similar to PA-NM-086, the two copies in PS2046 were present in tandem. The genetic elements were flanked by the same genes within the genomes of all three isolates (Fig. 3). C/T MICs among these isolates differed somewhat based on the antibiotic susceptibility test method (Table S3). PA-NM-086 and PS2046 were uniformly classified as resistant to C/T across methods. PS2045 exhibited higher C/T MICs with MBD vs MTS (Table S3). Additional copies of *bla*_L2_ were numerically though not significantly associated with higher C/T MICs (Pearson correlation coefficient, *r* = 0.73–0.82 on the log_2_ scale across MBD and MTS methods, *P* > 0.05).

### Impact of expression of *bla*_L2_ on C/T resistance in *P. aeruginosa*

We hypothesized that the gain of *bla*_L2_ increases C/T resistance in *P. aeruginosa*. To test this hypothesis, a plasmid construct p*bla*_L2_ was used to induce expression of *bla*_L2_ in PA14 and PAO1. Induction of *bla*_L2_ in both strains increased C/T MICs by more than two doubling (log_2_) dilutions vs the empty vector (EV) controls across different methods (Table 2; Table S3).

### Associations between the expression level of *bla*_L2_ and C/T resistance in *P. aeruginosa*

We reasoned that strains with higher *bla*_L2_ copy numbers may express more L2 β-lactamase, which would in turn lead to higher C/T MICs. To examine this, RT-qPCR was performed on PA-NM-086, PS2045, and PS2046. The expression levels of the *bla*_L2_ genes in these strains correlated with the *bla*_L2_ copy number (Fig. S1A). Notably, expression of *bla*_L2_ in PA14-p*bla*_L2_ under uninduced (C/T MIC 2 µg/mL) and induced (C/T MIC 16 µg/mL) conditions correlated with C/T resistance of PA14-p*bla*_L2_ (Fig. S1). However, PS2045 (C/T MIC 32 µg/mL) and PS2046 (C/T MIC 256 µg/mL) expressed *bla*_L2_ less than PA14-p*bla*_L2_ with IPTG induction (C/T MIC 16 µg/mL). Similarly, PS2046 had lower *bla*_L2_ expression than PA-NM-086 even though both strains had a C/T MIC of 256 µg/mL (Table 2; Table S3). Because overexpression of *bla*_AmpC_ has been linked to C/T resistance (14), we measured *bla*_AmpC_ expression but found similar *bla*_AmpC_ transcript levels in all strains (Fig. S1B). Furthermore, sequences of the *bla*_AmpC_ genes were found to be identical (PDC-35) in PS2045, PS2046, and PA-NM-086 (Table 2).

### Loss of L2 β-lactamase decreases C/T resistance in *P. aeruginosa*

To confirm that L2 β-lactamase is one of the mechanisms behind the observed C/T resistance in our *P. aeruginosa* isolates, the effect of deletion of the *bla*_L2_ gene on C/T MICs was examined. Deletion of the *bla*_L2_ gene in PA-NM-086 was not tractable, given the five copies carried. Since PS2045 contains only one copy of *bla*_L2_ (Fig. 3), it allowed the targeted deletion of approximately 50% of the *bla*_L2,_ open reading frame, including the region coding for the active site of L2 β-lactamase, to construct the mutant strain PS2045∆*bla*_L2_^act^. The C/T MIC of PS2045∆*bla*_L2_^act^ (0.5 µg/mL) was 64-fold (6 log_2_) lower vs the parental strain (32 µg/mL) (Table 2; Table S3). The disruption of *bla*_L2_ in PS2045 also reduced MICs (fourfold or ≥2 log_2_) of aztreonam, cefepime, and ceftazidime (Table 2). Whether disruption of the *bla*_L2_ gene influenced resistance to carbapenems could not be determined because the parental strain, PS2045, was highly susceptible to the carbapenems tested. Induction of the expression of *bla*_L2_ increased resistance of PA14 as well as PAO1 to aztreonam, cefepime, and ceftazidime by >8-fold, i.e., >3 log_2_ (Table 2), but had no impact on carbapenem MICs (Table 2).

### AmpR_L2_^tr^ does not regulate expression of the L2 β-lactamase in PA-NM-086

**Fig 4:**
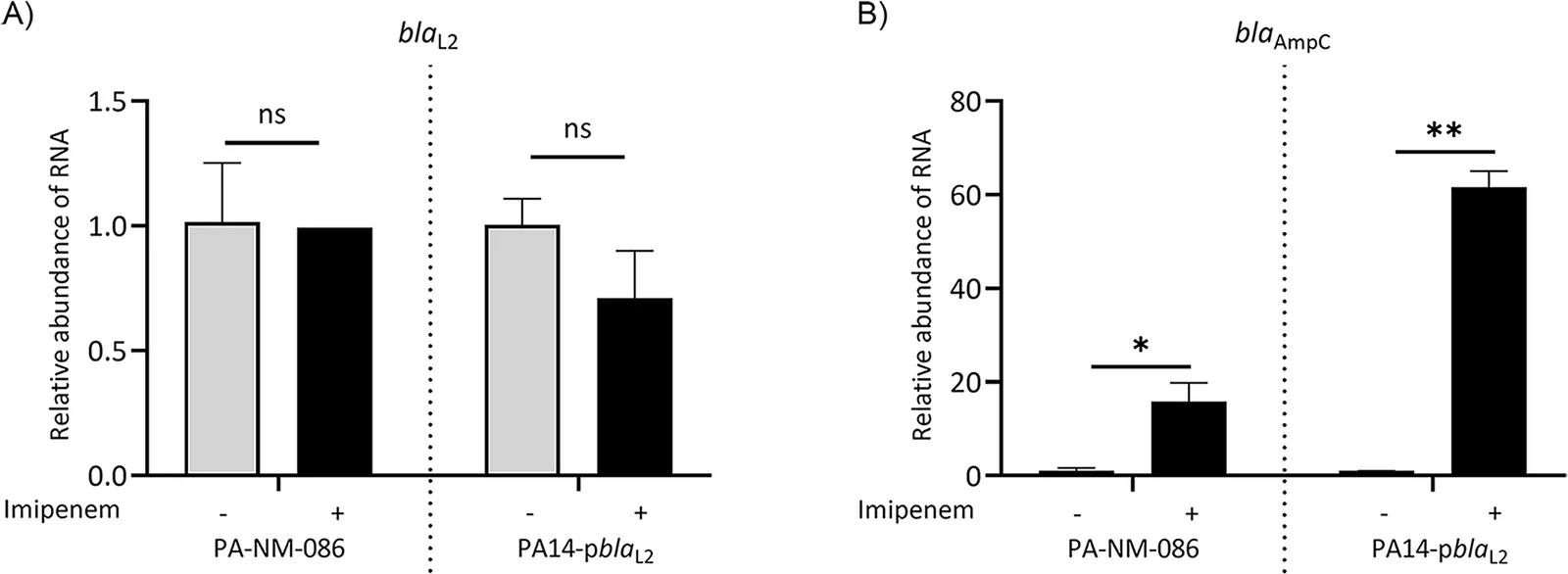
Expression of *bla*_L2_ and *bla*_AmpC_ in the presence and absence of imipenem. Expression of the genes (A) *bla*_L2_ and (B) *bla*_AmpC_ in the strains PA-NM-086 and PA14-*pbla*_L2_ in the presence (+) or absence (−) of imipenem was determined by RT-qPCR. Gene expression is shown as relative abundance in RNA levels in comparison to the respective untreated control (−) after normalizing to the housekeeping gene *rpoD*. The bars represent means of two biological replicates. Error bars represent standard deviations. Student’s *t*-test was used to determine statistical significance, indicated as *P* values, where ns, *P* > 0.05; *P < 0.05; and **P < 0.01.

As *ampR*_L2_^tr^ was truncated (867 bp reduced to 507 bp) in our isolates, we hypothesized that AmpR_L2_^tr^ does not regulate *bla*_L2_ expression. To test this hypothesis, the expression level of *bla*_L2_ in PA-NM-086 was evaluated in the presence and absence of the β-lactam imipenem that induces AmpR_L2_-dependent *bla*_L2_ expression in *S. maltophilia* (37). PA14-p*bla*_L2_ served as a negative control because *bla*_L2_ expression is independent of AmpR _L2_ in this strain. Expression of *bla*_AmpC_ induced by imipenem (38) served as a positive control. As expected, *bla*_L2_ expression in PA14-p*bla*_L2_ was induced by IPTG and not by imipenem (Fig. S1A and 4A), whereas *bla*_AmpC_ was induced by imipenem in PA-NM-086 as well as PA14-p*bla*_L2_ (Fig. 4B). In PA-NM-086, no significant difference was observed in *bla*_L2_ expression levels in the presence or absence of imipenem (Fig. 4A), indicating that *ampR*_L2_^tr^ does not regulate *bla*_L2_ expression. The deletion of *ampR*_L2_^tr^ in PS2045 did not affect the C/T MIC (Table 2).

## DISCUSSION

Our investigation provided several lines of evidence that suggest that the L2 β-lactamase of *S. maltophilia* acquired by *P. aeruginosa* contributed to C/T resistance. First, the *bla*_L2_-containing clinical isolates had elevated C/T MICs ranging from 32/4 to 256/4 µg/mL, similarly to *S. maltophilia* (39–41). Second, exogenous *bla*_L2_ expression in two distinct *P. aeruginosa* strains increased their C/T MICs. Third, the disruption of *bla*_L2_ caused a reduction in C/T MICs in PS2045. We also found that L2 β-lactamase contributed to resistance to multiple β-lactam antibiotics in *P. aeruginosa*, in a manner similar to that observed in *S. maltophilia* (37), but independently of the product of the *ampR*_L2_^tr^ gene. This latter finding may have been due to basal-level expression of *bla*_L2_ in the absence of AmpR_L2_, which has also been observed in *S. maltophilia* (42). The L2 β-lactamase, therefore, appears to be an example of cross-species transfer of antibiotic resistance mechanisms.

The 100% identity of the *bla*_L2_ gene sequences in *P. aeruginosa* and *S. maltophilia* and our genomic findings suggest that the *bla*_L2_ gene has been obtained from *S. maltophilia* along with a truncated *ampR*_L2_ gene as part of a 9,034 bp genetic element. The closely related strains PS2045, PS2046, and PA-NM-86 contained one, two, and five copies of this element, respectively, suggesting that selective pressures exist that caused repeated duplication of this element, possibly mediated by IS91 family transposases. However, duplication of *bla*_L2_ seems to be independent of C/T exposure, as PS2045 and PS2046 were collected in 2008, before C/T was FDA approved (1, 2). Notably, our patient had no history of colonization by *S. maltophilia* prior to the culture of PA-NM-086. L2 is a chromosomal class A serine β-lactamase in *S. maltophilia* (30). Hence, it is less likely to be readily transferred across species than the genes encoded on plasmids, which may explain why *bla*_L2_ was identified in only a handful of *P. aeruginosa* genomes. Both *P. aeruginosa* and *S. maltophilia* are ubiquitous environmental organisms found in soil, plant material, rivers, lakes, and streams (43, 44) and are cultured from individuals with cystic fibrosis (45), which may offer opportunities for genetic exchange.

This is the second report of the presence of an L2 β-lactamase gene in *P. aeruginosa*, the first being the acquisition of the *bla*_PME-1_ gene, which is similar to *bla*_L2_ (46). However, several observations suggest that the *bla*_PME-1_ gene is distinct from *bla*_L2_. PME-1 has only 50% amino acid identity to L2 β-lactamase of *S. maltophilia* (46), which is substantially less than the 68% amino acid identity observed between two distinct β-lactamases, SHV-1 and TEM-1. The *bla*_PME-1_ gene is carried on a 9-kb plasmid and was not adjacent to an *ampR*_L2_ gene. Although PME-1 conferred resistance to third-generation cephalosporins, including ceftazidime, it was inhibited by tazobactam and would therefore be predicted to be inactive against C/T. Furthermore, the *P. aeruginosa* strains that expressed L2 β-lactamase had elevated resistance to cephalosporins and monobactams, as also reported in *S. maltophilia* (30, 47), but remained susceptible to CZA, which agrees with the fact that serine β-lactamases are inactivated more efficiently by avibactam than tazobactam (30, 47–50). We therefore suggest that PME-1 and L2 β-lactamase be considered distinct β-lactamases.

Prior research has suggested that a common resistance mechanism to C/T among *P. aeruginosa* involves mutations leading to overexpression of (15, 17) or structural changes in the class C β-lactamase AmpC (PDC) involved in peptidoglycan recycling (4, 14, 19–22, 33, 51–60). Studies comparing isolates collected pre- and post-C/T treatment have found that alterations within the *bla*_AmpC_ gene are major drivers of increased C/T resistance (4, 15, 61). However, the *bla*_AmpC_ genes present in PA-NM-086, PS2046, and PS2045 were all identified as PDC-35, which does not contain the mutations that alter the activity of AmpC, such as F147L, ΔL223-Y226, E247K, and N373I (15, 17). Furthermore, PDC-35 does not significantly enhance C/T resistance upon exogenous expression in *P. aeruginosa* (17). Additionally, the *bla*_AmpC_ gene was expressed at similar levels in PA-NM-086, PS2046, and PS2045, which did not correlate with C/T MICs. Hence, it is unlikely that structural changes or overexpression of *bla*_AmpC_ was directly responsible for the elevated C/T MICs observed in these strains.

The reasons for the very high C/T resistance levels observed in PA-NM-086 and PS2046 in comparison to PS2045 remain unclear. Our results suggest that the L2 β-lactamase partially contributed to these high levels. However, the copy number of *bla*_L2_ genes had a modest and non-significant correlation with C/T MIC, suggesting the presence of additional mechanisms of resistance. Interestingly, PA-NM-086 contains a frameshift mutation in the gene *oprD*, which encodes the major porin OprD. A non-functional OprD may explain carbapenem resistance in PA-NM-086 (62), but its impact on C/T resistance is unclear (63). The ESBL OXA-488, a class D β-lactamase commonly found in C/T-resistant ST235 isolates of *P. aeruginosa* (61), and mutations in transcriptional regulators MexZ (Q103L) and NalC (E153Q), which can impact the function of MexXY and MexAB efflux pumps, respectively (64, 65), were identified in all three isolates, PA-NM-086, PS2045, and PS2046. Thus, these mutations failed to fully explain the observed C/T MICs in these isolates. None of the strains possessed the β-lactamases GES-6, SHV-2a, VIM-2, and IMP-28, which enhance resistance of *P. aeruginosa* to C/T (32, 57, 59, 66). Therefore, we speculate that additional unknown mechanisms contributed to the high C/T resistance observed in PA-NM-086 and PS2046.

Our study has several limitations. First, because we were unable to delete all five copies of the *bla*_L2_ gene in PA-NM-086, we were not able to ascertain what portion of PA-NM-086 C/T resistance was due to the L2 β-lactamase. Second, we lacked an antecedent C/T-susceptible strain of the same lineage as PA-NM-086 from our patient to strengthen a comparative genomics analysis. Third, our copy number experiments and deletion-expression analyses used different strains of *P. aeruginosa*, so we were unable to determine the effects of strain-specific background differences on C/T resistance. Fourth, the induction of expression of *bla*_L2_ in PA14 and PAO1 did not result in the same numerical C/T MIC as observed in PS2046, which expressed less *bla*_L2_ than PA14-p*bla*_L2_ with IPTG induction. However, the log-scale changes in C/T MIC were similar upon exogenous expression and deletion of *bla*_L2_, suggesting that the L2 β-lactamase can have a measurable impact on C/T resistance even at low copy numbers. Our analysis correlating *bla*_L2_ copy number with C/T MIC is hypothesis generating due to the low frequency of *bla*_L2_ presence in *P. aeruginosa*, which constrained our sample size.

### Conclusion

In conclusion, mutational and expression analyses suggest that acquisition of the class A β-lactamase gene *bla*_L2_ contributed to C/T resistance in *P. aeruginosa*. Additional studies are needed to better understand the processes that select for the transfer of resistance genes between *P. aeruginosa* and *S. maltophilia*. This study reports a rare but important case of antibiotic resistance that extends our understanding of C/T resistance in *P. aeruginosa*.

## Data Availability

The source data associated with the manuscript are available on the Prism repository of Northwestern University and can be accessed through the digital object identifier https://doi.org/10.18131/wdmvf-60k23.

## SUPPLEMENTAL MATERIAL

Supplemental material aac.00356-26-s0001.pdf Fig. S1; Tables S1 to S3.

Supplemental methods aac.00356-26-s0002.pdf Antibiotic susceptibility testing; whole-genome sequencing and bioinformatics; phylogenetic analysis; construction of strains expressing blaL2; generation of deletion mutants.

ASM does not own the copyrights to Supplemental Material that may be linked to, or accessed through, an article. The authors have granted ASM a non-exclusive, world-wide license to publish the Supplemental Material files. Please contact the corresponding author directly for reuse.
