# Supplementary material for: L2 β-lactamase contributes to ceftolozane-tazobactam resistance in Pseudomonas aeruginosa
Source: Antimicrob Agents Chemother. 2026 Sep 4;70(10):e00356-26. doi: 10.1128/aac.00356-26 (PMC13602457; doi:10.1128/aac.00356-26)
Supplement: Supplementary file 1 [file aac.00356-26-s0001.pdf]

Supplemental Data

Title: L2 β-lactamase contributes to ceftolozane-tazobactam resistance in *Pseudomonas aeruginosa*

Authors: Preeti Garai, PhD [1], Sophia H. Nozick, BS[1], Caroline C. Jozefczyk, PharmD[2*], Hannah Nam, MD, MSc[3,4], J. Nicholas O'Donnell, PharmD, MSc[5], Egon A. Ozer, MD, PhD[6], Alan R. Hauser, MD, PhD[1,6], Nathaniel J. Rhodes, PharmD, MSc[7,8,9#]

Affiliations: [1]Department of Microbiology-Immunology, Northwestern University, Feinberg School of Medicine, Chicago, IL, USA; [2] Midwestern University College of Pharmacy Downers Grove Campus, Downers Grove, IL and Department of Pharmacy, Northwestern Medicine, Chicago, IL, USA; [3]Stanford University School of Medicine, Stanford CA, USA; [4]School of Medicine, University of California Irvine, Irvine, CA, USA; [5]Department of Pharmacy Practice, Albany College of Pharmacy and Health Sciences, Albany, NY, USA; [6]Division of Infectious Diseases, Department of Medicine, Northwestern University Feinberg School of Medicine, Chicago, IL, USA; [7]Department of Pharmacy Practice, Midwestern University College of Pharmacy Downers Grove Campus, Downers Grove, IL, USA; [8]Midwestern University College of Pharmacy Downers Grove Campus, Pharmacometrics Center of Excellence, Downers Grove, IL, USA; [9]Department of Pharmacy, Northwestern Medicine, Chicago, IL, USA.

#Corresponding author and reprints: Nathaniel J. Rhodes, PharmD, MSC, BCPS AQ-Infectious Diseases, Associate Professor of Pharmacy Practice. Department of Pharmacy Practice, Midwestern University College of Pharmacy Downers Grove Campus. 555 31st Street, Downers Grove, IL. 60515; Office: AH364, 630-515-7376; Fax: 630-515-6958; Email: nrhode@midwestern.edu

*__Present address:__ Department of Pharmacy, Prisma Health – Greenville Memorial Hospital, Greenville, South Carolina, USA

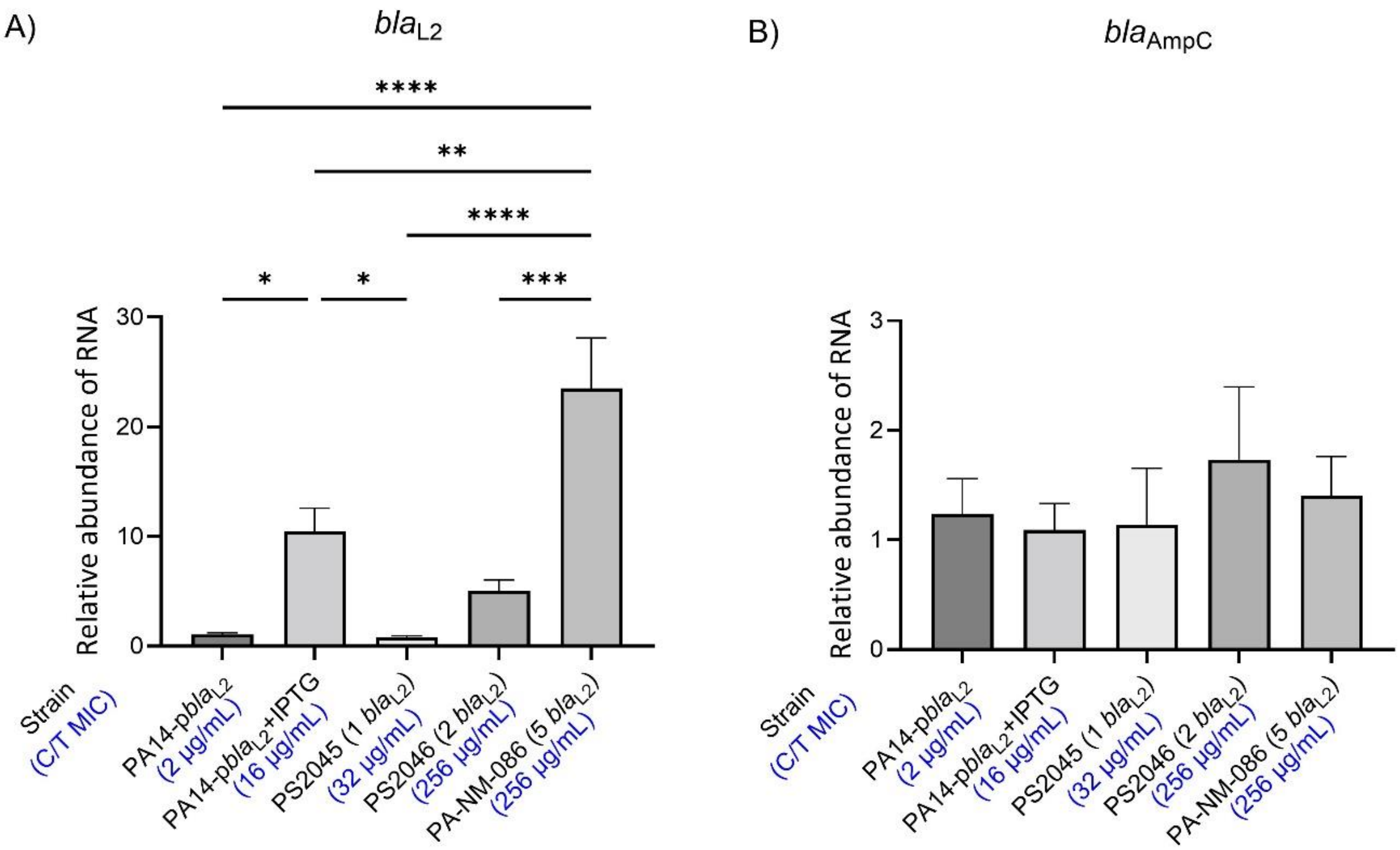

Fig. S1. *bla*$_{L2}$ expression in strains containing different *bla*$_{L2}$ copy numbers. Expression of the genes **A)** *bla*$_{L2}$ and **B)** *bla*$_{AmpC}$ in the strains PA14-p*bla*$_{L2}$ (with and without IPTG), PS2045, PS2046 and PA-NM-086 was determined by RT-qPCR. The number of *bla*$_{L2}$ genes is indicated in parentheses and the C/T MIC values of the strains in liquid medium are indicated in blue. Gene expression is shown as relative abundance in RNA levels with respect to PA14-p*bla*$_{L2}$ without IPTG after normalizing to *rpoD*. The bars represent mean of at least 2 biological replicates. Error bars represent standard deviation. One-way ANOVA was used to determine statistical significance and indicated as *p* values, * = *p* <0.05, ** = *p* <0.01, *** = *p* <0.001, **** = *p* <0.0001. Abbreviations- IPTG, isopropyl-β-D-thiogalactopyranoside.

Table S1. Primers and plasmids used for cloning and generating gene deletions are listed.

**Primer**	**Sequence**
pPSV37 EcoRI *bla*$_{L2}$ Fwd	5’ gattacgaattgtagctagctaggATGCTCGCCCGTCGCCGATTCC 3’
pPSV37 EcoRI *bla*$_{L2}$ Rev	5’ gaggatccccgggtaccgagctcgTCATCCGATCAACCGGTCG 3’
bla$_{L2}$ internal Fwd	5’ GCGACAACACCGCCGCCAACC 3’
bla$_{L2}$ internal Rev	5’ GCGGGCGGGCCACCGACCACG 3’
pPSV37 internal Fwd	5’ CAGGAAACAGCTATGACCAT 3’
pPSV37 internal Rev	5’ GCGATCAAAAAACCCCTCAA 3’
PS2045 *bla*$_{L2}^{act}$ Fwd up	5’ gtaaaacgacggccagtgccaGGATGTATGCGCGCGCCGAACC 3’
PS2045 *bla*$_{L2}^{act}$ Rev up	5’ aactcgagccgcaagcatgctgaaGCAGTGACCGCGTTCCTGC 3’
PS2045 *bla*$_{L2}^{act}$ Fwd down	5’ ttcagcatgcttgcggctcgagttCTTGATCGGGGGCGACCAGC 3’
PS2045 *bla*$_{L2}^{act}$ Rev down	5’ gagtcgacctgcaggcatgcaCTCATCGCGCGACCCTAGC 3’
PS2045 *bla*$_{L2}^{act}$ KO confirmatory Fwd	5’ GTTGGCCAGTTCCGTCAGTTGC 3’
PS2045 *bla*$_{L2}^{act}$ KO confirmatory Rev	5’ GCGCACATAGTTGGCGTGATAG 3’
PS2045 *ampR*$_{L2}^{tr}$ Fwd up	5’ gtaaaacgacggccagtgccaGTGACCGGTGCATGCGAGAGC 3’
PS2045 *ampR*$_{L2}^{tr}$ Rev up	5’ aactcgagccgcaagcatgctgaaGCTGGAGTGATGGATGATCC 3’
PS2045 *ampR*$_{L2}^{tr}$ Fwd_down	5’ ttcagcatgcttgcggctcgagttCGACCACCTGTTGCCGGTGC 3’
PS2045 *ampR*$_{L2}^{tr}$ Rev down	5’ gagtcgacctgcaggcatgcaGCAGCAGGATCGTGACGATC 3’
PS2045 *ampR*$_{L2}^{tr}$ KO confirmatory Fwd	5’ GGTGTTGTCGCTGGTGATGATC 3’
PS2045 *ampR*$_{L2}^{tr}$ KO confirmatory Rev	5’ CTTGGCCGCATCGGTCCAGC 3’
**Plasmid**	**Characteristics (Reference)**
pPSV37-Gen^R	Exogenous expression vector, Gen^R, *colE1* origin, PA origin, *oriT*, *lacUV5* promoter, *lacIq*, stops in every reading frame preceding the MCS and T7 terminator following the MCS (relative to the lacUV5 promoter) (1)
pEX18-Hyg^R	Allelic exchange vector, Hyg^R, *oriT*, *sacB*, *lacZα*, MCS from pUC18 (2)

Footnotes: **Primers** used for cloning indicate the plasmid and restriction site in their names and those used for gene deletion indicate the strain name and the target sequence. Cloning was verified with internal primers and gene deletion was verified with knockout (KO) confirmatory primers (see methods). All primer sequences have the region homologous to the gene amplified highlighted in capital letters. **Plasmid** names are shown with their marker antibiotic resistance genes (Gen- Gentamicin; Hyg- Hygromycin) and characteristics of the plasmids are shown with the source (reference) of the plasmid.

Table S2. RTqPCR primers and probes.

**Primer/Probe**	**Sequence**
*rpoD*_qPCR FWD	5’GGGCGAAGAAGGAAATGGT 3’
*rpoD*_qPCR REV	5’CTGGATCAGGTCGAGGAATTG 3’
*rpoD*_qPCR PRB	5’TCCATCGCCAAGAAGTACACCAACC 3’
bla$_{L2}$_qPCR FWD	5’ATCATCACCAGCGACAACAC 3’
bla$_{L2}$_qPCR REV	5’CCCTTGGCAAAGCTGTTCA 3’
bla$_{L2}$_qPCR PRB	5’AACCTGCTGTTCGGCGTGGT 3’
bla$_{AmpC}$ qPCR FWD	5’GATGCTCGGGTTGGAATAGAG 3’
bla$_{AmpC}$ qPCR REV	5’TCGACCTCGCGACCTATAC 3’
bla$_{AmpC}$ qPCR PRB	5’TGCAGAAGGACCAGGCACAGATC 3’

Footnotes: Forward (FWD) and Reverse (REV) primers, and probes (PRB) used for determining expression of the genes *rpoD*, *bla*$_{L2}$ and *bla*$_{AmpC}$ are listed with their sequences.

Table S3. Ceftolozane-Tazobactam (C/T) resistance measured by different methods.

C/T MICs (µg/mL)			
**Strain**	**MIC Test Strip (MTS)**	**Microbroth dilution (MBD)**	**Antibiotic-plate (Sensititre)**
PA-NM-055	0.75/4	¼	<=2/4
PA-NM-086	>256/4	256/4	>=32/4
PS2046	>256/4	256/4	>=32/4
PS2045	**4/4**	**32/4**	**16/4**
PS2045 Δbla_{L2}^{act}	0.5/4	0.5/4	<=2/4
PS2045 $\Delta ampR_{L2}^{tr}$	**4/4**	**32/4**	**16/4**
PA14-EV	0.125/4	0.25/4	NA
PA14-pbla_{L2}	**0.19/4**	**2/4**	NA
PAO1-EV	0.5/4	0.5/4	NA
PAO1-pbla_{L2}	0.75/4	0.5/4	NA
PA14-EV + IPTG	0.125/4	0.25/4	<=2/4
PA14-pbla_{L2}+ IPTG	**0.75/4**	**16/4**	**8/4**
PAO1-EV + IPTG	0.38/4	0.5/4	<=2/4
PAO1-pbla_{L2}+ IPTG	**3/4**	**32/4**	**>=32/4**

Footnotes: C/T MICs (µg/mL) for strains used in the study are listed as determined by MTS, MBD and Sensititre. The median or higher of 2 medians of MIC values from 2 or more experiments was chosen as the final MIC. C/T MICs that varied across methods of assessment are highlighted in bold. NA indicates results not available.

References:

1. Lee PC, Stopford CM, Svenson AG, Rietsch A. 2010. Control of effector export by the Pseudomonas aeruginosa type III secretion proteins PcrG and PcrV. Mol Microbiol 75:924-41.

2. Hoang TT, Karkhoff-Schweizer RR, Kutchma AJ, Schweizer HP. 1998. A broad-host-range Flp-FRT recombination system for site-specific excision of chromosomally-located DNA sequences: application for isolation of unmarked Pseudomonas aeruginosa mutants. Gene 212:77-86.
