# Supplementary material for: L2 β-lactamase contributes to ceftolozane-tazobactam resistance in Pseudomonas aeruginosa
Source: Antimicrob Agents Chemother. 2026 Sep 4;70(10):e00356-26. doi: 10.1128/aac.00356-26 (PMC13602457; doi:10.1128/aac.00356-26)
Supplement: Supplementary file 2 [file aac.00356-26-s0002.pdf]

Supplemental Methods

Title: L2 β-lactamase contributes to ceftolozane-tazobactam resistance in *Pseudomonas aeruginosa*

Authors: Preeti Garai, PhD [1], Sophia H. Nozick, BS[1], Caroline C. Jozefczyk, PharmD[2*], Hannah Nam, MD, MSc[3,4], J. Nicholas O'Donnell, PharmD, MSc[5], Egon A. Ozer, MD, PhD[6], Alan R. Hauser, MD, PhD[1,6], Nathaniel J. Rhodes, PharmD, MSc[7,8,9#]

Affiliations: [1]Department of Microbiology-Immunology, Northwestern University, Feinberg School of Medicine, Chicago, IL, USA; [2] Midwestern University College of Pharmacy Downers Grove Campus, Downers Grove, IL and Department of Pharmacy, Northwestern Medicine, Chicago, IL, USA; [3]Stanford University School of Medicine, Stanford CA, USA; [4]School of Medicine, University of California Irvine, Irvine, CA, USA; [5]Department of Pharmacy Practice, Albany College of Pharmacy and Health Sciences, Albany, NY, USA; [6]Division of Infectious Diseases, Department of Medicine, Northwestern University Feinberg School of Medicine, Chicago, IL, USA; [7]Department of Pharmacy Practice, Midwestern University College of Pharmacy Downers Grove Campus, Downers Grove, IL, USA; [8]Midwestern University College of Pharmacy Downers Grove Campus, Pharmacometrics Center of Excellence, Downers Grove, IL, USA; [9]Department of Pharmacy, Northwestern Medicine, Chicago, IL, USA.

#Corresponding author and reprints: Nathaniel J. Rhodes, PharmD, MSC, BCPS AQ-Infectious Diseases, Associate Professor of Pharmacy Practice. Department of Pharmacy Practice, Midwestern University College of Pharmacy Downers Grove Campus. 555 31st Street, Downers Grove, IL. 60515; Office: AH364, 630-515-7376; Fax: 630-515-6958; Email: nrhode@midwestern.edu

*__Present address:__ Department of Pharmacy, Prisma Health – Greenville Memorial Hospital, Greenville, South Carolina, USA

Antibiotic Susceptibility Testing

For microbroth dilution (MBD) susceptibility testing on Sensititre plates (Thermo Fisher Scientific, Waltham, Massachusetts, US), isolates were grown overnight on Luria-Bertani (LB) agar at 37°C. The following day, 3-5 colonies were picked up and re-suspended in water and diluted to an optical density at 600 nm (OD_{600}) of 0.07-0.10 that corresponded to ~10^8 CFU/mL (0.5 McFarland). As per manufacturer's instructions, a total of 30 µL of the bacterial suspension was inoculated into 11 mL of cation-adjusted Mueller-Hinton Broth (CA-MHB). Wells were inoculated with 50 µL of the suspension, and the negative control was inoculated with 50 µL of CA-MHB only. The plate was incubated at 37°C for 20 hours before reading.

MBD assays performed by hand followed a protocol adapted from Clinical and Laboratory Standards Institute (CLSI) guidance (1). Ceftolozane (Sigma SBR00048, Saint Louis, MO, USA) was dissolved in water to 2048 µg/mL and then serially diluted 2-fold in CA-MHB to a final concentration of 0.25 µg/mL. Tazobactam (Sigma, PHR1686, Laramie, WY, USA) dissolved in water and diluted in CA-MHB to 16 µg/mL was added to each TOL stock to obtain C/T concentrations ranging from 1024/8 to 0.125/8 µg/mL. Strains grown on LB agar were resuspended in CA-MHB to achieve ~10^8 CFU/mL as described above. The suspension was further diluted to ~5×10^5 CFU/mL in CA-MHB, and 50 µL (~2.5×10^4 CFU) was added to 50 µL of each C/T solution in a 96-well microtiter plate, such that the final C/T concentration ranged 512/4 to 0.0625/4 µg/mL. The plate was incubated for 16 hours at 37°C before reading.

MIC test strip (MTS) assays were performed according to manufacturer protocol (Liofilchem, Roseto degli Abruzzi, Italy). Briefly, strains were streaked onto LB agar plates and grown overnight at 37°C. Individual colonies were resuspended in CA-MHB to achieve an OD_{600} corresponding to ~10^8 CFU/mL as described

above. The suspension was plated onto CA-MHA using cotton swabs. GDS containing C/T were placed on the plates and incubated for 16-20 hours at 37°C.

For all MBD and MTS assays, *P. aeruginosa* strain ATCC 27853 was used as quality control. For all MBD experiments, the lowest antibiotic concentration resulting in no visible growth was the MIC. For all MTS experiments, MICs were defined according to manufacturer's instructions as the intersection of bacterial growth ellipse with the indicated concentration on the strip. For vector construct and empty vector control strains, C/T MIC experiments were carried out using media containing gentamicin (15 µg/mL) with or without 1 mM IPTG.

Whole Genome Sequencing and Bioinformatics

Isolates from frozen stocks (-80°C) were streaked onto Luria-Bertani (LB) agar plates, and a single colony was cultured in LB broth overnight at 37°C. Genomic DNA was extracted using the Promega Maxwell 16 Cell DNA Purification Kit (Promega, Madison, WI, USA). Libraries were prepared using the Nextera XT DNA Library Preparation Kit (Illumina, Inc., San Diego, CA, USA) and sequenced on a NextSeq 500 instrument (150 base pair reads, paired-end, mid-output flow cell, 133 M read pairs). Sequences obtained as short reads were trimmed using Trimmomatic v0.32 (2) and then *de novo* assembly was performed with SPAdes 3.9.1 (3). Contigs shorter than 200 bp or with an average read coverage of less than 5x were excluded. For long-read sequencing, libraries were prepared from separate cultures grown on different days using the Oxford Nanopore ligation sequencing kit SQK-LSK110 and sequenced on a FLO-MIN106 flow cell on a MinION sequencing device. Guppy v3.4.5 was used to base call reads with the R9.4.1 high-accuracy model. Assembly of long reads was performed using the Trycycler v0.5.0 pipeline (4). Reads were filtered using

Filtlong v0.2.1 to remove reads shorter than 1000 bases and the bottom 5% of the reads based on quality. Reads were then subsampled into 24 subsets using the Trycycler "subsample" function. Eight read subsets were assembled using Flye v2.8.3 (5), eight read subsets were assembled using Raven v1.5.3, and eight read subsets were assembled using minimap2 v2.21, miniasm v0.3, and minipolish v0.1.3 (6, 7). Clustering, reconciliation, circularization, multiple sequence alignment, and consensus sequence generation from the resulting 24 assemblies were performed using Trycycler. The consensus assembly was polished with medaka v1.4.3 using the "r941_min_hac_g507" model. Illumina reads were aligned to the assembly using BWA v0.7.17 (8), and assembly errors were corrected using Pilon v1.23 (9) with a minimum depth setting of 0.1. Read alignment and Pilon correction were performed sequentially until no further assembly corrections were identified. Polypolish v0.4.3 (10) was used for final assembly correction using the Illumina short reads. Sequencing reads and genome assemblies are deposited in NCBI under BioProject accession PRJNA547625. The *bla*$_{L2}$ gene deletion mutant in strain PS2045 was sequenced using a commercial service (SEQCENTER, Pittsburgh, PA, USA) to produce paired-end 150 bp reads. Sequencing reads were aligned to the complete genome assembly of PS2045 and assessed for mutations relative to the reference using Breseq v0.39.0 (11).

Phylogenetic analysis

All sequence type 235 genomes (n=2996, accessed Oct 14, 2025) of the *P. aeruginosa* genome assemblies (n=46,236, accessed Oct 14, 2025) in the NCBI assembly database were downloaded. One outlier genome was dropped and whole genome phylogenetic relationships were estimated for 2995 ST235 genomes using Mashtree v1.2.0 (12). The presence of the *bla*$_{L2}$ gene sequence was identified in the genome sequences using nucleotide BLAST. Ten ST235 genome assemblies without a full-length *bla*$_{L2}$ gene were selected randomly. Illumina read sequences were downloaded from NCBI for all L2-containing sequences

identified by nucleotide BLAST against the NCBI WGS database with results limited to *P. aeruginosa* (taxid 287). For the four ST235 isolate sequences for which sequence reads were not available in the Sequence Read Archive (SRA) database of NCBI, pseudoreads were generated from the genome assemblies as previously described (13). Short read and pseudoread sequence alignment, phylogenetic analysis, and tree visualization were performed as previously described (14).

Construction of strains expressing bla_{L2}

The gene bla_{L2} was amplified from the genomic DNA of PA-NM-086 using Q5 high fidelity polymerase (New England Biolabs, Ipswich, MA, USA) and primers 1 and 2 (**Table S2**). The amplicon was integrated at the EcoRI site downstream to *lac*UV5 promoter in the plasmid pPSV37-Gen^R (15) by Gibson assembly (Thermo Fisher Scientific Baltics UAB, Lithuania). This plasmid does not contain an ampicillin resistance marker and therefore does not add to the β-lactam resistance level of the recipient strain. The resulting recombinant plasmid "pbla_{L2}" was introduced into the conjugative *E. coli* strain SM10 (16) by the calcium chloride heat shock method, and the transformants were selected on LB agar containing gentamicin (15 µg/mL). Conjugation was performed to transfer the recombinant plasmid from the transformant SM10 strain to the *P. aeruginosa* strains, and the exconjugants were selected on LB agar containing gentamicin (30 µg/mL) and irgasan (5 µg/mL). The plasmid construct was isolated (Qiagen, Germantown, MD, USA) from the exconjugant strains and verified by Sanger sequencing using internal primers recognizing the bla_{L2} gene and an adjacent plasmid pPSV37 sequence (**Table S2**). Vector control strains were constructed by introducing the empty vector pPSV37 into PA14 and PAO1 (PA14-EV and PAO1-EV) using the same method.

RNA extraction and reverse-transcriptase-quantitative-PCR (RT-qPCR)

Overnight cultures of bacterial strains grown in LB medium were subcultured in LB medium, grown to an OD_{600} of 0.4, and treated with IPTG (1 mM) or imipenem (1 µg/mL) for additional 2 hours to test the induction of gene expression in the indicated experiments. Negative control cultures were left untreated. A volume of the culture corresponding to 1 x 10^8 CFU was centrifuged, and the pellet was resuspended in RNA protect reagent (Qiagen, Germantown, MD, USA). After incubation for 5 min at room temperature, samples were centrifuged again, and bacterial cells in the pellet were treated with lysozyme (15 µg/mL in Tris-EDTA). After incubation for 5 min at room temperature, samples were centrifuged again, and bacterial cells in the pellet were treated with lysozyme (15 µg/mL in Tris-EDTA). RNA was extracted using an RNEasy kit (Qiagen, Germantown, MD, USA), and cDNA was synthesized with Superscript IV VILO reverse transcriptase kit (Invitrogen, Thermo Fisher Scientific Baltics UAB, Lithuania) using master mix with and without reverse transcriptase, the latter used as a negative control to assess for DNA contamination. RT-qPCR was performed using qPCR probe assays (Integrated DNA Technologies, Corallville, IA, USA) on a CFX-96 real-time thermocycler (Biorad). The threshold cycle (Ct or Cq) value of the housekeeping gene *rpoD* ($Ct_{reference}$) was subtracted from Ct value of the target gene (Ct_{target}) for every sample generating ΔCt values. The ΔCt values of the control strain PA14-p*bla*$_{L2}$ without IPTG treatment were subtracted from corresponding ΔCt values of all strains to obtain ΔΔCt values. Finally, the $2^{-\Delta\Delta Ct}$ values were plotted to indicate gene expression levels as relative abundance of RNA. The primers and probes used for the RT-qPCR experiments are listed in **Table S3**. Each experiment was carried out at least twice (i.e., at least 2 biological replicates).

Generation of deletion mutants

P. aeruginosa strain PS2045 was used as the background strain to generate gene deletion mutants using a suicide-vector mediated allelic replacement method (17). Fwd-up and Rev-up primers were used to

generate a ~500 bp fragment homologous to 5' end ('up' fragment), whereas Fwd-down and Rev-down primers were used to generate a ~500 bp fragment homologous to 3' end ('down' fragment) of the target genes bla_{L2} and $ampR_{L2}^{tr}$ (**Table S2**). However, a 936 bp transposon gene immediately 3' to the bla_{L2} gene in PS2045 is duplicated elsewhere in the genome of PS2045, preventing amplification of an 'up' fragment. To avoid the duplicated region, we targeted deletion of a 477 bp internal segment of bla_{L2} instead of the entire gene. This segment encodes the S-X-X-K motif and SDN loop, which are required for the activity of the L2 β-lactamase (18). The 'up' and 'down' fragments were combined using the Fwd-up and Rev-down primers to generate the full fragment, which was integrated at the HindIII site of the plasmid pEX18-Hyg^{R} (17) by Gibson assembly (Thermo Fisher). The resulting construct was introduced into the conjugative *E. coli* strain SM10 (16) by calcium chloride heat shock method. The transformants were selected on LB agar containing hygromycin (100 µg/mL) and used for conjugation with the strain PS2045 at 37°C for 1 hour. Resulting merodiploids with the entire plasmid integrated into the genome were selected using LB agar supplemented with hygromycin (500 µg/mL) and irgasan (5 µg/mL). Counter-selection of merodiploids using LB agar supplemented with sucrose (10 ug/mL) yielded mutants that lost the *sacB*-containing vector backbone along with the target gene. The deletion of the target gene in the selected colonies was verified by PCR amplification with confirmatory primers (**Table S2**) followed by Sanger sequencing of the PCR product. Genomic DNA from mutant isolates was sequenced using a commercial service (SEQCENTER) to produce paired-end 150 bp reads. Sequencing reads were aligned to the complete genome assembly of isolate PS2045 and assessed for mutations relative to the reference using Breseq v0.39.0 (11).

References:

1. Wiegand I, Hilpert K, Hancock RE. 2008. Agar and broth dilution methods to determine the minimal inhibitory concentration (MIC) of antimicrobial substances. Nat Protoc 3:163-75.
2. Bolger AM, Lohse M, Usadel B. 2014. Trimmomatic: a flexible trimmer for Illumina sequence data. Bioinformatics 30:2114-20.
3. Prjibelski A, Antipov D, Meleshko D, Lapidus A, Korobeynikov A. 2020. Using SPAdes De Novo Assembler. Curr Protoc Bioinformatics 70:e102.
4. Wick RR, Judd LM, Cerdeira LT, Hawkey J, Meric G, Vezina B, Wyres KL, Holt KE. 2021. Trycycler: consensus long-read assemblies for bacterial genomes. Genome Biol 22:266.
5. Kolmogorov M, Yuan J, Lin Y, Pevzner PA. 2019. Assembly of long, error-prone reads using repeat graphs. Nat Biotechnol 37:540-546.
6. Li H. 2018. Minimap2: pairwise alignment for nucleotide sequences. Bioinformatics 34:3094-3100.
7. Wick RR, Holt KE. 2019. Benchmarking of long-read assemblers for prokaryote whole genome sequencing. F1000Res 8:2138.
8. Li H. 2013. Aligning sequence reads, clone sequences and assembly contigs with BWA-MEM. arXiv:13033997
9. Walker BJ, Abeel T, Shea T, Priest M, Abouelliel A, Sakthikumar S, Cuomo CA, Zeng Q, Wortman J, Young SK, Earl AM. 2014. Pilon: an integrated tool for comprehensive microbial variant detection and genome assembly improvement. PLoS One 9:e112963.
10. Wick RR, Holt KE. 2022. Polypolish: Short-read polishing of long-read bacterial genome assemblies. PLoS Comput Biol 18:e1009802.
11. Deatherage DE, Barrick JE. 2014. Identification of mutations in laboratory-evolved microbes from next-generation sequencing data using breseq. Methods Mol Biol 1151:165-88.

12. Katz LS, Griswold T, Morrison SS, Caravas JA, Zhang S, den Bakker HC, Deng X, Carleton HA. 2019. Mashtree: a rapid comparison of whole genome sequence files. J Open Source Softw 4.

13. Santoiemma PP, Cohn SE, Gatesy SWM, Hauser AR, Agrawal S, Theodorou ME, Bachta KER, Ozer EA. 2024. The global population stru cture of Lacticaseibacillus rhamnosus and its application to an investigation of a rare case of infective endocarditis. PLoS One 19:e0300843.

14. Atassi G, Medernach R, Scheetz M, Nozick S, Rhodes NJ, Murphy-Belcaster M, Murphy KR, Alisoltani A, Ozer EA, Hauser AR. 2023. Genomics of Aminoglycoside Resistance in Pseudomonas aeruginosa Bloodstream Infections at a United States Academic Hospital. Microbiol Spectr 11:e0508722.

15. Lee PC, Stopford CM, Svenson AG, Rietsch A. 2010. Control of effector export by the Pseudomonas aeruginosa type III secretion proteins PcrG and PcrV. Mol Microbiol 75:924-41.

16. Simon R, Priefer, U. & Pühler, A. . 1983. A Broad Host Range Mobilization System for In Vivo Genetic Engineering: Transposon Mutagenesis in Gram Negative Bacteria. Nature Biotechnology 1:784–791.

17. Hoang TT, Karkhoff-Schweizer RR, Kutchma AJ, Schweizer HP. 1998. A broad-host-range Flp-FRT recombination system for site-specific excision of chromosomally-located DNA sequences: application for isolation of unmarked Pseudomonas aeruginosa mutants. Gene 212:77-86.

18. Walsh TR, MacGowan AP, Bennett PM. 1997. Sequence analysis and enzyme kinetics of the L2 serine beta-lactamase from Stenotrophomonas maltophilia. Antimicrob Agents Chemother 41:1460-4.
